# Changes in the Invasion Rate of *Prosopis juliflora* and Its Impact on Depletion of Groundwater in the Northern Part of the United Arab Emirates

**DOI:** 10.3390/plants11050682

**Published:** 2022-03-02

**Authors:** Fares M. Howari, Manish Sharma, Yousef Nazzal, Ali El-Keblawy, Shajrat Mir, Cijo M. Xavier, Imen Ben Salem, Ahmed A. Al-Taani, Fatima Alaydaroos

**Affiliations:** 1 College of Natural and Health Sciences, Zayed University, Abu Dhabi P.O. Box 144534, United Arab Emirates; fares.howari@zu.ac.ae (F.M.H.); yousef.nazzal@zu.ac.ae (Y.N.); mir.shajrat@gmail.com (S.M.); cijo.xavier@zu.ac.ae (C.M.X.); imen.bensalem@zu.ac.ae (I.B.S.); ahmed.al-taani@zu.ac.ae (A.A.A.-T.); 2 Department of Applied Biology, College of Sciences, University of Sharjah, Sharjah P.O. Box 27272, United Arab Emirates; akeblawy@sharjah.ac.ae; 3 UAE Space Agency, Abu Dhabi P.O. Box 7133, United Arab Emirates; f.alaydaroos@space.gov.ae

**Keywords:** evapotranspiration, groundwater, invasive alien species, *Prosopis* invasion, remote sensing

## Abstract

*Prosopis* species were introduced to the United Arab Emirates (UAE) region for desert greening. However, the species now pose a great threat to the native plant diversity. This study used high-resolution satellite imagery (1990–2019) to understand the history and current distribution of *Prosopis* species and their impact on fresh groundwater. The results show that the *Prosopis* invasion in the study area reached its maximum expansion rate in 2019 and covered an area of about 16 km^2^ compared to 0.2 km^2^ in 1990. The areas near Sharjah Airport, Umm Fannan, and Al Talla, located at a lower elevation of the sand dune area, are heavily invaded. *Prosopis* groundwater requirement derived using evapotranspiration shows that groundwater consumption has changed drastically after 2010 and consumed about 22.22 million m^3^ of groundwater in 2019, which is about a 7372% increase in groundwater consumption from the year 1990 to 2019. The results can be useful for setting up a management plan for the sustainable use of this species in the UAE region in particular and other similar countries in the arid land regions that are suffering from freshwater depletion because of *Prosopis* invasion.

## 1. Introduction

The groundwater in the United Arab Emirates (UAE) is limited and deteriorating due to overconsumption in the agricultural and industrial sectors. Moreover, the hot arid climate that receives limited rainfall, that evaporates, experiences a large amount of evapotranspiration that exacerbates the problem of groundwater resources in the UAE [1]. The achievement of a deeper understanding of the dynamics of invasive woody plant encroachment to determine its expansion rate and detrimental impact on fresh groundwater is a task of pivotal importance in the United Arab Emirates. Russell et al. [2] quantified the patterns of hydraulic redistribution by mesquite and assessed how this affects tree water use and productivity. They found that *Prosopis juliflora* (mesquite) switches between shallow lateral and deep taproots, which allow them to extract more groundwater. Further, the hydraulic redistribution would greatly enhance the moisture for the *Prosopis* plants. The increase in the number of introduced invasive species can change the social–ecological systems of a particular region [3,4,5].

*Prosopis* species are xerophytes that can thrive in harsh desert environments [6]. They can also grow in semi-arid, saline–alkaline soils where no other plant species except xerophytes grow. The ability of the plant to grow on sand dunes with scarce vegetation indicates that it can combat desertification and mitigate the adverse effects of climate change [7]. It is also used as timber, fuelwood, shelter, building materials, and furniture for local farmers in many regions. *Prosopis* plants can produce large amounts of non-dormant seeds that are capable of long-distance dispersal, thus spreading over a large area [8,9,10]. The biological invasion by *P. juliflora* is recognized as a primary threat to indigenous biodiversity [11]. Several mechanisms have been proposed for the successful invasion of exotic trees, including *P. juliflora.* These include the production of allelochemicals, shade effect, and competition for water and nutrients [12,13,14]. Dzikiti et al. [15] reported a hydraulic redistribution under *Prosopis* as evidenced by the apparent increase in the soil water content at the 75 and 150 cm depths during summer, affecting the species abundance and diversity. In addition, Dalle Fratte et al. [16] indicated that invasive alien and native plant species occupy the same niches and have the same requirements, indicating that competitor alien plants can replace the native species. Furthermore, Van Kleunen et al. [17] concluded that invasive alien species had higher values of traits related to performance (e.g., growth rate, size, and fitness) than native species, further supporting the ability of the alien to replace the native plants.

*Prosopis juliflora* is a native tree to North America (Mexico) or Central America (Costa Rica, El Salvador, Guatemala, Honduras, Nicaragua, Panama) [13,18]. It has been introduced to several counties in hot tropical and arid regions for several purposes, including dune stabilization, afforestation, providing fodder, fuelwood, and shade [13]. For example, *Prosopis juliflora* was introduced to Sudan early in the 19th century to combat desertification and as a source of fuelwood [19]. Similarly, it was brought to Lake Baringo, Kenya, in the 1980s to overcome the problem of fuelwood shortage [20]. *P. juliflora* was introduced to India by the end of the last century to reclaim sodic and salt-affected lands and as a source of fuelwood, timber, and fiber [21,22] and into Ethiopia in the 1970s for conserving soil and water resources [23]. The sale of charcoal and *Prosopis* pods for fodder have enhanced the local economy in some areas in Kenya by USD 1.5 million per year [24]. In India, *Prosopis* species have provided up to 70% of fuelwood for local households in some dry region villages [22]. Although these plant species provide some benefits, the problems caused due to their invasive nature are far more severe. The IUCN has considered *P. juliflora* as one of the world’s worst 100 invasive alien species [25]. In addition, *P. juliflora* was considered among 18 alien plants with high priority for a pest risk analysis for the European list of invasive alien plants [26].

In the UAE, *P. juliflora* was introduced during the 1970s to combat desertification in Abu-Dhabi Emirate [27]. However, as seeds of *P. juliflora* can be disseminated by domestic livestock such as goats, cattle, mules, camels, and wild fauna such as gazelles [28,29,30,31], it invaded large areas in the northern and western Emirates, such as Ras Al-Khaimah, Fujairah, Ajman, Sharjah, Dubai, and Umm Al Quwain [32]. The higher rainfall in addition to shallower groundwater in the northern and western Emirates make these regions even more suitable for the invasion of *P. juliflora* [33,34]. Recent studies have reported deterioration of native plants due to *Prosopis* invasions resulting in a significant reduction in the area’s biodiversity, negatively impacting local flora and fauna. Issa et al. [35] have also reported the aggressive invasive characteristics of *Prosopis* species in parts of the UAE. They used high-resolution digital aerial photographs, to assess the change and evaluate mesquite’s invasion dynamic in two sites of the Northern Emirates, UAE, for three different decades (1986, 1996, and 2005). The study concluded that the spread of mesquite across the Northern Emirates is significantly increasing at an alarming rate [35]. In addition, Garrido et al. [36] used water relations and foliar isotopic composition to study the *Prosopis* plant at three levels of water table depth. The Eddy covariance method was used to study the evapotranspiration of *P. julifora* and its impacts on the catchment water budget in the Afgar region of Ethiopia [37]. Moreover, a comparison of water uses and groundwater implication of *Prosopis* spp. and the associated *Vachellia karroo* trees were conducted by [38] in South Africa. Moreover, the impacts of *Prosopis* on groundwater level and water use was carried out in South Africa [39] and Abu Dhabi [40]. However, remote sensing technology-based studies have not been reported anywhere.

**Figure 1:**
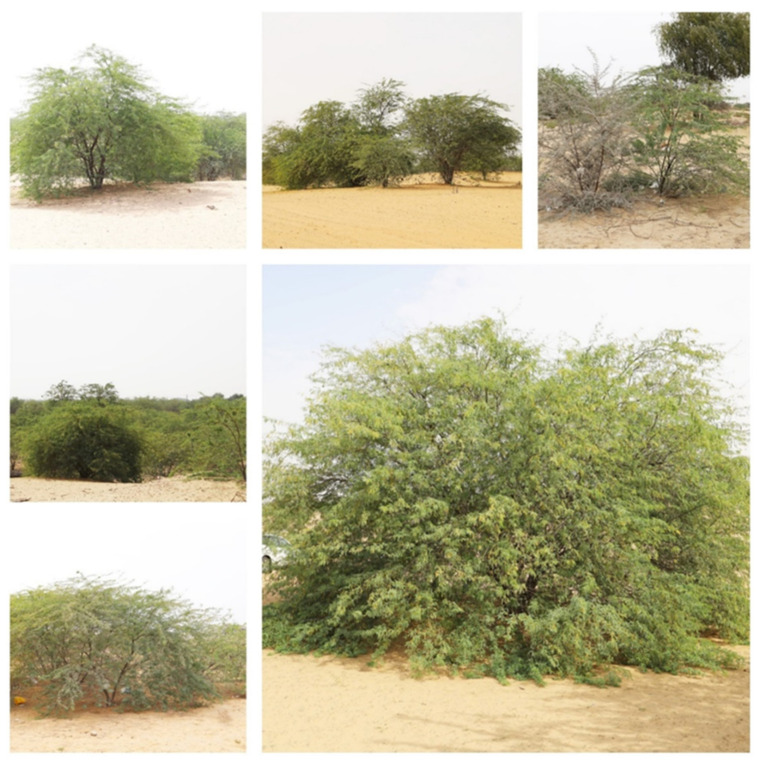
Mesquite tree (*Prosopis juliflora*) in Ajman. Photos show competition between the exotic *P. juliflora* and the native *P. cineraria* in the right and middle photos of the upper row and the left photo of the middle row.

The focus of this study is to calculate changes in coverage of this species for over 30 years by using Landsat series remote sensing satellite data and its invasive impact on fresh groundwater declination. This study monitored the spread and propagation of the invasive *P. juliflora* in newly invested areas in the Ajman and Sharjah Emirates. We integrated geographic information system (GIS), remote sensing images, and meteorological station data to detect and quantify the rate of invasion around the most affected area in Ajman Emirate from the early 1990s to 2019. We also calculated its impact on groundwater by estimating evapotranspiration using Sharjah Airport meteorological station data. The mesquite tree (*Prosopis*) images in the Ajman Emirates are shown in Figure 1. Some images show the association and dense competition between the exotic *P. juliflora* and the native *P. cineraria*.

## 2. Materials and Methods

### 2.1. Study Area

**Figure 2:**
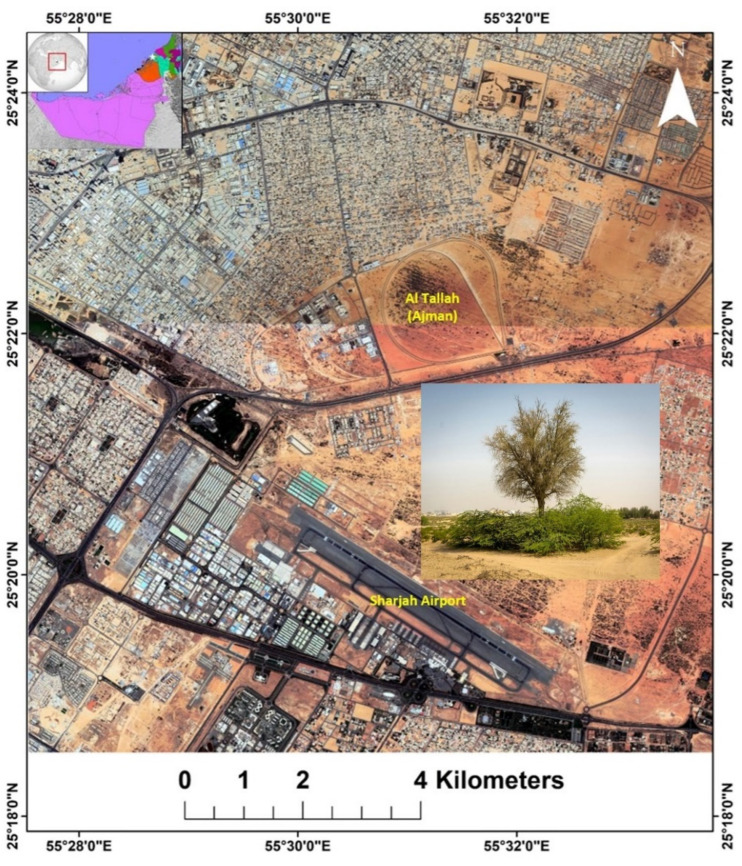
Map of the investigated study area showing the invasive *P. juliflora* site. Inset image showing densely growing *P. juliflora* competing with the native *P. cineraria*, which results in death of native trees.

The study area is located near a sand dune area between Al Ramanih, Sharjah and Al Talla, Ajman and extends between longitude 55°29′24″ to 55°36′36″ E and latitude 25°18′36″ to 25°23′24″ N as shown in Figure 2. Sand dunes cover the study area with small portions of built-up areas and farms. The area is surrounded by Al Hellio farms in the north, the built-up area in the east and the northwest, and the Sharjah Airport in the south. The UAE climate is arid to hyper-arid, characterized by low rainfall and high temperatures with high relative humidity [41]. Table 1 shows the temperature and precipitation data collected from the nearest meteorological station in the study site at Sharjah Airport for the year 2019.

**Figure 3:**
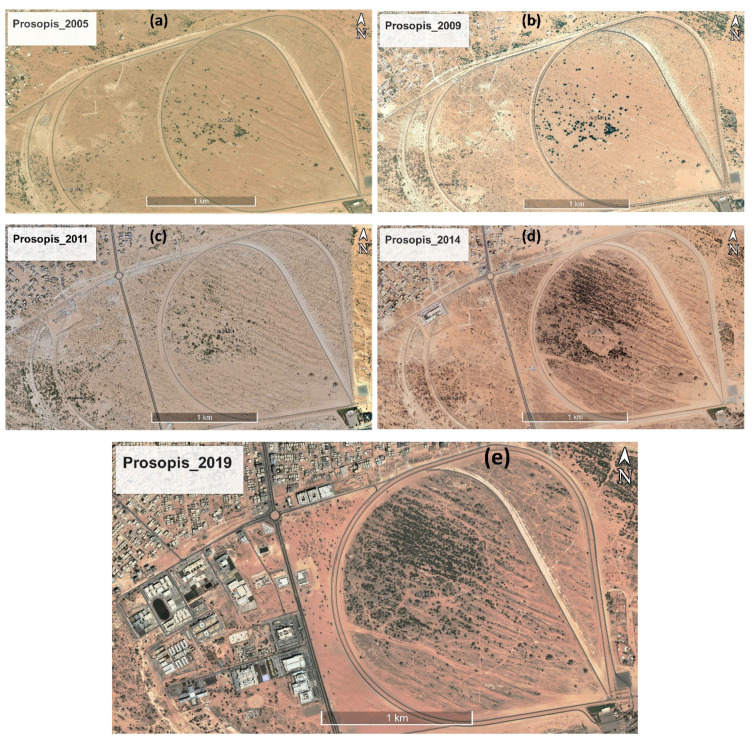
The satellite images collected from Google Earth Pro show the invasion of *P. juliflora* in sand dunes near Al Tallah Camel Racecourse, UAE (**a**) 2005, (**b**) 2009, (**c**) 2011, (**d**) 2014, and (**e**) 2019.

There are two main seasons: summers are harsh and dry, and winters have mild-to-warm temperatures. Rainfall is erratic and unpredictable in time and quantity all over the peninsula. In general, most rainfall happens in December–February [42]. Before the invasion of *P. juliflora*, these sandy dunes had scarce vegetation. The satellite images from Google Earth Pro are used to show a significant spread of mesquite in the area around Al Tallah racecourse, Ajman Emirate of the UAE, in Figure 3.

The scarce vegetation of the study area is characterized by small xerophytic shrubs, herbs, and a few numbers of trees. Slate et al. [43] recorded the accumulation of a great amount of litter under and around the canopies of *P. juliflora* plants. Such litters inhibited the growth of most native plants, reducing the plant diversity and abundance. *Prosopis cineraria* is the native plant most affected by the growth of *P. juliflora* plants [43]. The trees of *P. cineraria* have special importance for the local environment and people of the UAE. It facilitates the environment in terms of the growth of several native species and provides food for animals and people [44]. The dispersal of the exotic *P. juliflora* seeds through grazing animals under the canopy of the native *P. cineraria* encourages the high-density growth of the exotic under the native *Prosopis*.

### 2.2. Data Collection

Multispectral and hyperspectral remote sensing data are important in the prior phases of exploration, particularly in arid and semi-arid regions [45]. In the current study, a set of multi-temporal and multi-source remote sensing data was collected from USGS Earther Explorer website to monitor and analyze mesquite (*P. juliflora*) changes over three decades. This includes four ortho-rectified cloud-free remote sensing images with dates 23 August 1990 (Landsat 4 satellite data), 23 August 2000 (Landsat 4–5 TM satellite data), 6 September 2010 (Landsat 7 ETM+ satellite data), and 14 April 2019 (Landsat 8 OLI), respectively.

Field data was acquired using a spectroradiometer and Trimble GPS in the preliminary stage of the project. Satellite data verification was completed using acquired field investigated GPS coordinates. The field site provided the training data used for supervised and feature (*P. juliflora*) extraction classification for the remote sensing images. They also provided data for accuracy assessment [46]. In the current study, *P. juliflora* canopy reflectance measurement was undertaken using a SVC Field Spectroradiometer. The SVC HR-768si is unmatched for high-resolution field measurements between 350 and 1900 nm, setting a new standard within the remote sensing community.

Landsat derived normalized difference vegetation index (NDVI) values were validated using field photographs and Quick Bird images obtained from Google Earth. This approach demonstrated an effective use of the remote sensing data and spatial analysis for vegetation studies: a combination of digital satellite images, GIS cartographic tools, and methods of spatial analysis of vegetation coverage are highly suitable and efficient for the monitoring of highly heterogeneous landscapes located in an area of intensive anthropogenic activities [47].

### 2.3. Data Analysis

The Landsat images were geo-processed for the detection of changes in the land cover types using ArcGIS and ENVI software. Remotely sensed data were initially co-registered with higher precision using 45 ground control points (GCPs) collected from the Quick Bird image to avoid any misregistration. The obtained Landsat images had Root Mean Square Error (RMSE) values of 0.23, 0.31, 0.35, and 0.29 pixels, respectively, and these images were improved by applying the enhanced Lee filter [48].

The normalized difference vegetation index (NDVI) was derived using Landsat images to monitor and analyze changes in *Prosopis* from each image using the ArcGIS raster calculator tool. NDVI is a dimensionless index, which describes the difference between visible and near-infrared reflectance of vegetation cover and is commonly used to estimate the density of vegetation in a region. NDVI is a direct indication of vegetation vigor and the vegetation state of the region. NDVI was calculated using Landsat satellite data bands as follows:

NDVI = (NIR − RED)/(NIR + RED)

RED and NIR stand for the spectral reflectance measurements acquired in the red and near-infrared regions, respectively. The produced NDVI maps were converted into vector format to calculate the total area in km^2^ and the percentage for each map. The healthy and non-healthy *Prosopis* were discriminated based on the NDVI values, where dispersed *Prosopis* were shown to have a lower value (0.1–0.3), and dense *Prosopis* had high NDVI values (>0.3). Before monitoring *Prosopis* changes, an accuracy assessment was performed on each NDVI map using field observation. The image difference algorithm (ID) was applied to each pair of NDVI maps to monitor and detect *Prosopis* changes [49]. The resultant change detection map contains two different color codes: red highlights the changing area, while blue highlights areas with no visible change. To see the topographic difference in the study area, digital elevation model (DEM) data was generated from NASA’s Shuttle Radar Topography Mission (SRTM) using ESRI ArcGIS Software.

### 2.4. Estimation of Evapotranspiration and P. juliflora Groundwater Requirement

Water is becoming an increasingly limited resource due to climate change and increased demands from a growing human population. Proper water management to enhance water usage efficiency is critical in arid climates where freshwater resources are scarce. Evapotranspiration (ET) is the sum of evaporation and plant transpiration [50]. The concept of reference crop evapotranspiration (ET_o_) was introduced to study the evaporative demand of the atmosphere independently of crop type, crop development stage, and management practices [51,52]. The calculation of reference evapotranspiration is a very common method used to calculate the crop water requirement. There are several methods used to calculate or measure ET_o_. The most common methods are the Thornthwaite method and the Penman–Monteith method. The Thornthwaite method uses climate data that can be obtained from a weather station. To determine crop evapotranspiration (ETc), reference crop potential evapotranspiration (PET or ET_o_) and crop coefficient (K_c_) should be estimated. We used the Thornthwaite method to calculate the evapotranspiration (ET) in the study area by estimating the heat index using mean temperature of year 2019, as shown in Table 1. The Thornthwaite monthly ET_o_ is determined with the equation proposed by [53] for a standard month of 30 days and days with a 12 h photoperiod by using the monthly mean temperature.

Using the Thornthwaite method, we can calculate Potential Evapotranspiration (PET); first, the Monthly Thornthwaite Heat Index (i) is calculated, using the following formula:

i = (t/5)^1.514(1)

where t is the monthly average temperature.

The Annual Heat Index (I) is calculated as the sum of the Monthly Heat Indices (i):

(2) $$I=\overset{12}{\underset{i=1}{\sum }}i$$

A Potential Evapotranspiration (PET) estimation is obtained for each month, considering a month is 30 days long and there are 12 theoretical sunshine hours per day, applying the following equation:

PET_non corrected_ = 16 ×(10 × t/I)^α (3)

where α is

α = 675 × (10)^(−9) × I^3 − 771 × (10)^(−7) × I^2 + 1792 × (10)^(−5) × I + 0.49239(4)

Obtained values are later corrected according to the real length of the month and the theoretical sunshine hours for the latitude of interest, with the formula:

PET or ET_o_ = PET_non corrected_ × (N/12) × (d/30) (5)

N is the theoretical sunshine hours for each month and d number of days for each month.

The crop coefficient (K_c_) of *Prosopis* was found to be about 0.77 [25], which is used to calculate ET_crop_, which is evapotranspiration calculated using mesquite as the reference crop.

ET_crop_ = K_c_ × ET_o_ (6)

The crop water requirement (CWR) budget equation was used to calculate maximum groundwater consumption by *Prosopis*. The equation is described as follows:

Total Groundwater Used (m^3^) = ET_crop_ × A × 0.0254 (7)

where ET_crop_ is *P. juliflora* crop evapotranspiration, A is area (square meters), and 0.0254 is a conversion factor for ET (inch to meters).

## 3. Results and Discussion

### 3.1. Field Data: Identification of Mesquite (P. juliflora) Based on Reflectance

**Figure 4:**
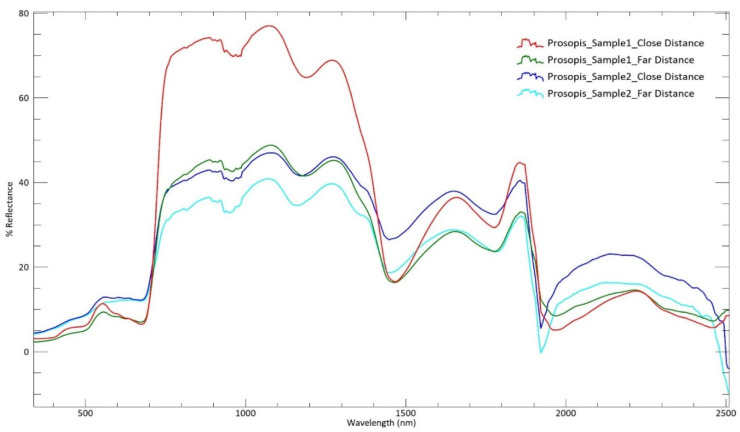
The field spectral reflectance of invasive species (mesquite) in a semi-arid area of Ajman.

Descriptive data and spectra were acquired during field research to analyze the main characteristics of *Prosopis* in Ajman. Each plant species possesses unique absorption features. In *P. juliflora*, broad/major absorption features were recorded at 679, 1440, 1769, and 1938 nm, while minor absorption features were at 416, 503, 976, and 1171 nm, respectively, as shown in Figure 4. All data recordings were obtained from the field spectroradiometer camera and were then decoded in ENVI software for the analysis. Figure 4 shows the spectral signatures of two different *P. juliflora*, having spectra recorded at close and far distances in the UAE region on 22 February 2019, particularly in the Ajman area (25°19′37″ N, 55°32′60″ E), acquired in the preliminary stage of the project.

### 3.2. Monitoring Prosopis Change

**Figure 5:**
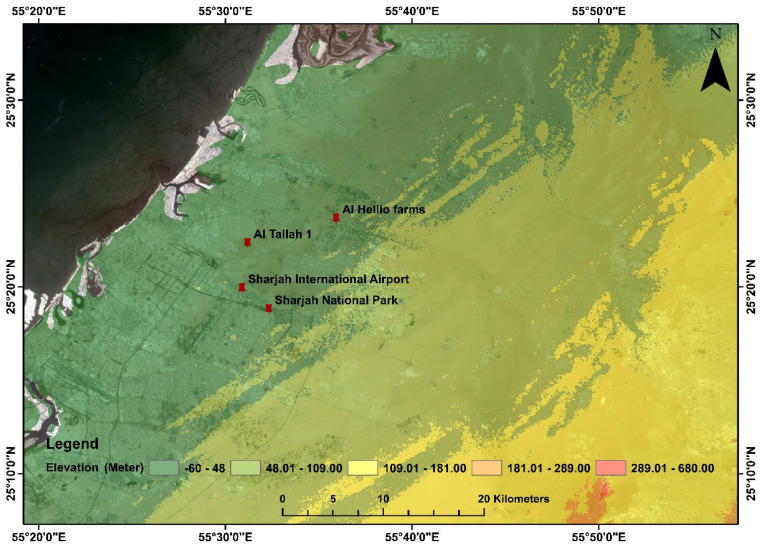
Digital elevation model map of the study area.

The results show that the areas near Sharjah Airport, Umm Fannan and Al Talla, located at a lower elevation of sand dune area are heavily invaded by *Prosopis* species and distributed in clusters and dispersed forms. Topography of the area can be observed in Figure 5, showing Al Tallah 1 region at 12 m elevation, whereas Al Hellio farms, and Sharjah International airport are observed at 30 and 57 m elevations.

The results also show that dispersed *Prosopis* slightly invade the area near Al Minzah (NE). The majority of *Prosopis* were found colonizing sand dune areas at a lower elevation, near the built-up, agricultural area, and water body. Some higher elevated areas along sand dune corridors are not affected like low elevation regions. Such results support our earlier findings that the growth of *P. juliflora* is better under the moist sandy sites than drier ones under the local conditions of the UAE [27,32].

**Figure 6:**
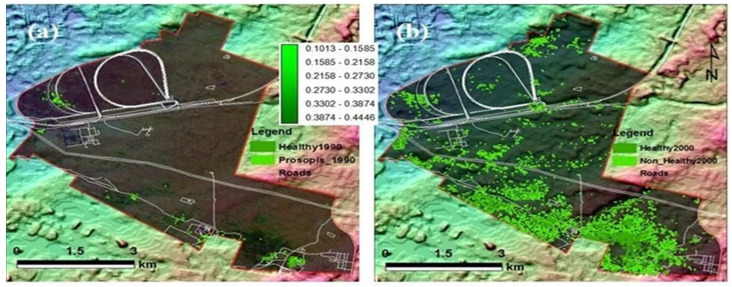

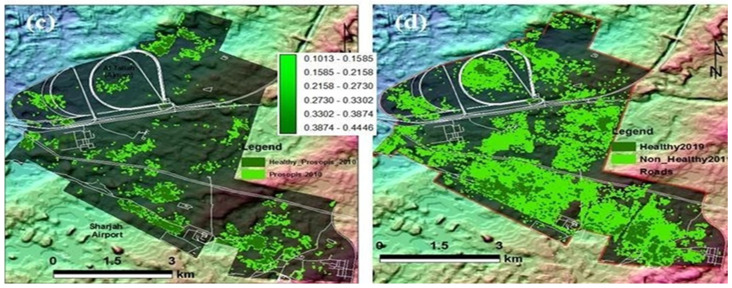
NDVI maps derived from Landsat series satellite for the years (**a**) 1990 (Landsat 4–5 TM), (**b**) 2000 (Landsat 4–5 TM), (**c**) 2010 (Landsat 7 ETM+), and (**d**) 2019 (Landsat 8 OLI), respectively. The background of the image is shown with the hill shade elevation effect.

In this study, the direction of *Prosopis* expansion was found in the NW–SE and WNW–ESE directions. The image processing results also indicate very few *Prosopis* in the area during 1990, as shown in Figure 6a. The estimated area was about 0.214 km^2^ (0.58%) of the total investigation site of 36.487 km^2^. In 1990, out of 0.214 km^2^ of *Prosopis*, 0.019 km^2^ (0.052%) was dense *Prosopis*. From 1990 to 2000, there was a significant increase in *Prosopis* growth from 0.214 (0.58%) to 5.21 km^2^ (14.55%). In the year 2010, the *Prosopis* distribution was observed to be 5.31 km^2^. Dakhil et al. [18] expected a global high invasion rate of *P. juliflora* with increasing temperature and soil alkalinity. The UAE climate is hot to very hot most of the year, and the soil pH is alkaline [27,32,54].

**Figure 7:**
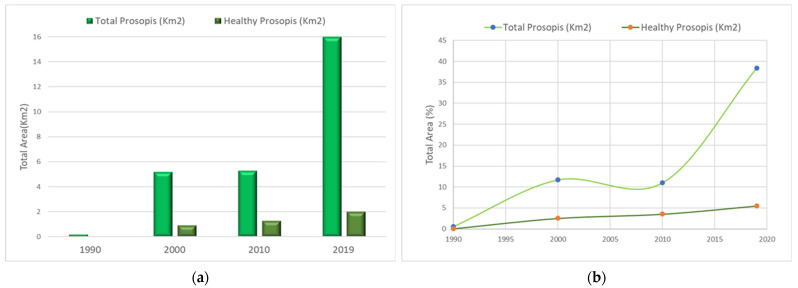

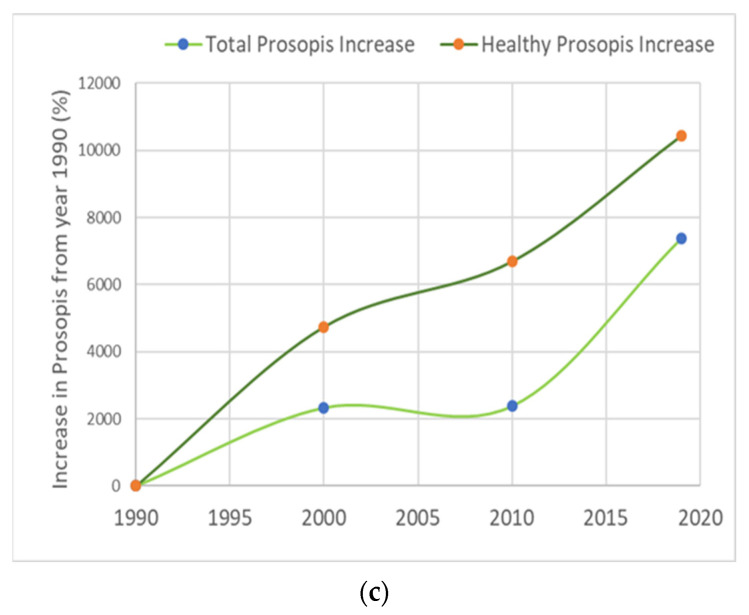
Graphical representation showing (**a**) *Prosopis* changes in km^2^, (**b**) with percentage of the area covered in the study site and (**c**) percentage increase in *Prosopis* from the year 1990 to 2010.

Since 2010, *Prosopis* continued to increase, reached its maximum rate in 2019, and covered an area of about 15.99 km^2^ (43.82%). In 2019, a notable clustering of *Prosopis* forests can be seen in the areas near Sharjah Airport, Umm Fannan, and Al Tallah, as shown in Figure 6d. There is a clear distinction between dense *Prosopis* and dispersed *Prosopis* areas in the NDVI maps. Dense *Prosopis* showed a high NDVI value (>0.3), while dispersed *Prosopis* showed a lower value (0.1–0.3) for NDVI. Figure 6 and Figure 7 and Table 2 show the *Prosopis* distribution in the study area.

The densest portions of *Prosopis* are mainly observed in Al Rahmanya (behind Sharjah Airport) and Al Talla (Ajman). Very few were found in Al Muntazi (northeast), next to Hamidiya, Ajman. Such a drastic increase in the density and sizes of the invasive *Prosopis* in 2019 could be explained by the large amounts of rainfall received in the UAE. It has been reported that March 2016 received 287 mm in 24 h [55]; the average annual rainfall in the study area is around 100 mm. The great amount of rainfall that occurred in March 2016 was explained by performing 77 seeding operations. This extraordinary rainfall reached the groundwater and enhanced the growth, especially in low-lying areas that received more water through runoff, such as Al Rahmanya and Al Talla.

### 3.3. Prosopis Change Detection

**Figure 8:**
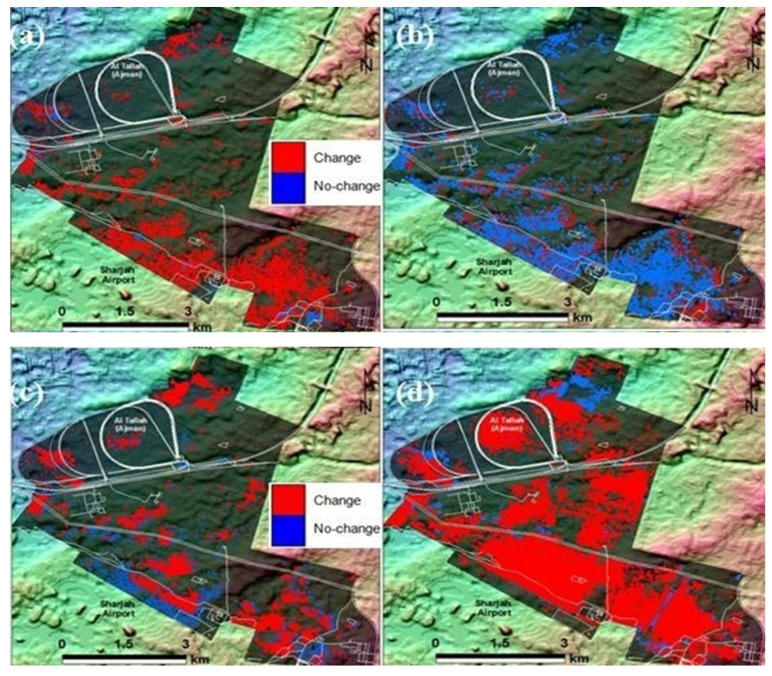
*Prosopis* change detection maps derived from a pair of classification maps using the image difference algorithm for years (**a**) 1990–2000, (**b**) 2000–2005, (**c**) 2005–2010, and (**d**) 2010–2019.

The maps of detection changes obtained by using the image difference algorithm are shown in Figure 8. The map consists of two-color codes. The red color highlights the changes in *Prosopis*, and the blue color indicates no change. The results obtained indicate that from 1990 to 2000, approximately 2.04 km^2^ (5.59%) of the study area was occupied by slight *Prosopis*. The assessment of *Prosopis* changes from periods 2000–2005 and 2010–2019 confirmed that net *Prosopis* area increases were more pronounced from 2010 to 2019 as compared to 2000–2005, rising from 3.19 (8.73%) to 11.08 km^2^ (30.36%), respectively. Positive change in healthy and non-healthy *Prosopis* was higher from 2010 to 2019 compared to the period from 2000 to 2005 and much higher compared to the period from 1990 to 2000, as shown in Figure 8. The areas coded with red color, which indicate a continuous increase in *P. juliflora*, are mainly in sandy areas subjected to great disturbance. The local environmental sectors in the municipalities of Ajman and Sharjah Emirates are continually eradicating the plants in areas with higher density. They usually remove the above crown, but not the superficial dense roots that reach more than 10 m away from the trunk. Such disturbance results in the stimulation of adventitious buds on the roots of the removed plants. In addition, soil disturbance brings buried seeds to the surface, enhancing germination and seedling emergence [8,9].

### 3.4. Evapotranspiration and P. juliflora Water

**Figure 9:**
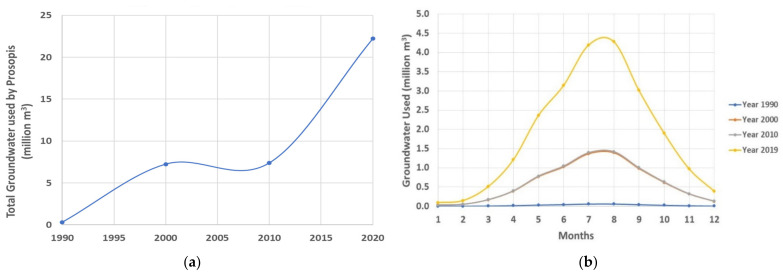
Graphical representation of (**a**) total groundwater consumption by *Prosopis* and (**b**) showing monthly total groundwater consumption variation by *Prosopis* (million m^3^) (1990–2019).

The calculation of reference evapotranspiration (ET_o_) is a common method to calculate the crop water requirement. Evapotranspiration calculated using mesquite as the reference crop (ET_c_) using meteorological data from 2019 for the investigated area is shown in Table 3. The *P. juliflora* groundwater requirement derived using evapotranspiration shows a very high increase from 1990 to 2019, as shown in Table 4 and Figure 9. The calculated groundwater for the year 1990 was about 0.297 million m^3^, for an area of about 0.214 km^2^ (0.58%) of the total investigated area. From 1990 to 2000, there was a significant increase in *Prosopis* growth, which consumed 2334% more groundwater making a total consumption of 7.239 million m^3^ of groundwater, for an increased area of 5.31 km^2^ (14.55%) of *Prosopis* invasion. *Prosopis* distribution changed drastically after 2010 and continued to spread over a large area, which consumed about 22.2197 million m^3^ of groundwater, which is about a 7372% increase in groundwater consumption from 1990 to 2019. The great water consumption of *P. juliflora* might affect the groundwater level and competition with native shrubs, reducing biological diversity [38]. In addition, the continued depletion of the groundwater might also reduce the future invasive ability of *Prosopis*. However, the future pattern of *Prosopis* invasion in the investigated site could also be affected by leaking water from agricultural farms in the surrounding areas (via subsurface channels). Future monitoring programs for the groundwater level and the native biodiversity are important to assess the risks associated with *Prosopis* invasion and deterioration of native plant diversity.

A previous study on *Prosopis* plants indicated a total groundwater consumption of 3.1–3.3 billion m^3^/year, using the Eddy covariance method in the Afar region (Ethiopia) [25]. Moreover, the consumption in Northern Cape (South Africa) was 70 m^3^/month, according to the Penman–Monteith methodology [27]. However, Al Yamani et al. [30] calculated water consumption of the two native trees (*Prosopis cineraria* and *Ziziphus spina-christi*) in Abu Dhabi, United Arab Emirates and found very low consumption (0.043 m^3^/day), using the single crop-factor approach. The current study used remote sensing technology and reported an estimate of 22.22 million m^3^/year in the studied region. Our results indicate that the multi-temporal and high-resolution remote sensing data captured from numerous satellites can examine the broad area of plant propagation in a distant and isolated area, having benefits over other techniques.

## 4. Conclusions

*Prosopis* distribution changed drastically after 2010 and spread over a large area, consuming about 22.219 million m^3^ of groundwater in 2019. The water consumption in 2019 was about 7372% that of 1990. *Prosopis* consumed around 0.06 million m^3^ of water per day in the invaded areas in 2019. Hence, this fast-growing exotic tree is likely to exacerbate the effects of climate change on the provision of ecosystem services through its impact on the ecosystem water budget. The groundwater depletion in the study area, mainly dunes, will threaten its xerophytic scarce vegetation, especially the native keystone *P. cineraria*.

Our study has added to the growing body of evidence regarding how rapidly mesquite has invaded the UAE, particularly in Ajman and Sharjah areas, and the negative impacts of this expansion on the UAE’s natural ecosystems and the services they deliver. We applied NDVI to multiple sources of optical remote sensing data collected using Landsat series imagery. The healthy and non-healthy *Prosopis* were discriminated based on the positive NDVI generated map values, where dispersed *Prosopis* were shown to have a lower value (0.1–0.3), and dense *Prosopis* had high NDVI values (>0.3). The distinct invasive pattern of *Prosopis* in the study area can be affected by leaking water from agricultural farms in the Al Hellio area into the *Prosopis* area located at a lower elevation, through seawater intrusion (via subsurface channels) or by grazing in the study area. The growing evidence presented in this study and elsewhere of the widespread negative impacts of *Prosopis* invasions will continue to increase unless a solution can be found. Although *Prosopis* has some benefits for the Bedouin campsites, it has a serious negative impact on groundwater quantity in the area. Thus, removing and controlling the densest *Prosopis* in the study area is recommended. We recommend that a full assessment of the costs and benefits be carried out to inform policy decisions. Creating national strategic plans would also help guide management and prevent inefficiency in the future.

## Figures and Tables

**Table 1:** Monthly variation in average temperature and precipitation in the nearest meteorological station in the study site at Sharjah Airport in 2019. (Source: Cimate-data.org, accessed on 21 February 2020).

Parameter	January	February	March	April	May	June	July	August	September	October	November	December
Avg. Temp (°C)	18	18.9	21.5	24.4	28.2	30.6	33	33.5	31.2	27.7	23.8	20.1
Prec. (in)	0.7	0.7	0.7	0.2	0	0	0.1	0	0	0	0.1	0.4

**Table 2:** Estimates of *Prosopis* classes total area and percentage and changes from 1990 to 2019. Here, % refers to the area coverage of *Prosopis* in the investigated site (36.487 km^2^), and % increase refers to % increase in *Prosopis* from the year 1990.

Year	1990		2000			2010			2019		
Parameters	km^2^	%	km^2^	%	% Increase	km^2^	%	% Increase	km^2^	%	% Increase
*Prosopis*	0.195	0.53	4.29	11.7	2100	4.02	11.0	1961	13.99	38.36	7075
Healthy *Prosopis*	0.019	0.052	0.92	2.53	4742	1.29	3.54	6689	2.00	5.49	10,426
*Prosopis* Total Area	0.214 km^2^		5.21 km^2^		2334%	5.31 km^2^		2381%	15.99 km^2^		7371%

**Table 3:** Monthly reference evapotranspiration (ET_o_) and mesquite crop evapotranspiration (ET_c_) were calculated using the Thornthwaite method for 2019 (Evapo_Groundwater showing the total evapotranspiration by *Prosopis*, calculated by subtracting the precipitation value from *P. juliflora* crop evapotranspiration).

Month	ET_o_ mm/month	ET_crop_ mm/month	ET_crop_ inch/month	Precipitation (inch)	Evapo_Groundwater
January	30.77	23.69	0.932	0.7	0.232
February	35.12	27.04	1.064	0.7	0.364
March	64.76	49.86	1.963	0.7	1.263
April	105.28	81.06	3.191	0.2	2.991
May	192.70	148.37	5.841	0.0	5.841
June	256.60	197.58	7.779	0.0	7.779
July	345.97	266.40	10.488	0.1	10.388
August	349.68	269.25	10.600	0.0	10.600
September	246.28	189.64	7.466	0.0	7.466
October	155.50	119.73	4.714	0.0	4.714
November	82.60	63.60	2.504	0.1	2.404
December	44.85	34.54	1.359	0.4	0.959
Annually	1910.16	1470.82	57.906	2.9	55.006

**Table 4:** Total groundwater used by *P. juliflora* from 1990 to 2019 (million m^3^).

Month	1990	2000	2010	2019
January	0.0013	0.0306	0.0312	0.0940
February	0.0020	0.0480	0.0489	0.1473
March	0.0068	0.1663	0.1695	0.5103
April	0.0162	0.3937	0.4013	1.2084
May	0.0316	0.7689	0.7836	2.3597
June	0.0421	1.0239	1.0435	3.1423
July	0.0562	1.3673	1.3935	4.1963
August	0.0573	1.3952	1.4220	4.2821
September	0.0404	0.9827	1.0015	3.0159
October	0.0255	0.6205	0.6324	1.9043
November	0.0130	0.3164	0.3225	0.9712
December	0.0052	0.1263	0.1288	0.3877
Annually	0.2974	7.2398	7.3787	22.2197

## Data Availability

Data will be available on request.
